# Particularities of the *Hermetia illucens* (L.) (Diptera: Stratiomyidae) Ovipositing Behavior: Practical Applications

**DOI:** 10.3390/insects13070611

**Published:** 2022-07-07

**Authors:** Georgescu Bogdan, Struți Dănuț Ioan, Șuteu Mihai, Moldovan Lavinia Elena, Moldovan Dorin Vasile, Boaru Anca Mihaela

**Affiliations:** 1 Department of Zoology and Ecology, Faculty of Animal Science and Biotechnologies, University of Agricultural Sciences and Veterinary Medicine Cluj-Napoca, 400372 Cluj-Napoca, Romania; georgescu.bogdan63@yahoo.com; 2 Department of Technological Science, Faculty of Animal Science and Biotechnologies, University of Agricultural Sciences and Veterinary Medicine Cluj-Napoca, 400372 Cluj-Napoca, Romania; suteu_usamv@yahoo.com (Ș.M.); lavinia.muntean@usamvcluj.ro (M.L.E.); 3 Department of Geotechnical and Foundations, Faculty of Construction, Technical University of Cluj-Napoca, 400144 Cluj-Napoca, Romania; dorin.moldovan@dst.utcluj.ro

**Keywords:** reproductive behavior, egg clutch, black soldier fly, oviposition

## Abstract

**Simple Summary:**

The industrial rearing of *Hermetia illucens* is based on knowledge of the reproductive particularities and reproductive behavior, which are important for increasing the efficiency of the rearing technology. Therefore, this study aimed to evaluate the particularities of the ovipositing behavior of females from the perspective of their affinity towards the size of the ovipositing slots, the correlations between this and the resulting egg mass, as well as the effect of the humidity of attractive substrate on the oviposition particularities. Over 90% of *H. illucens* females oviposited with predilection for ovipositing devices with slots of 1–3 mm. The differentiated affinity for oviposition in 1-, 2- and 3-mm slots, depending on the size of the females, is positively correlated with the size of the resulting clutches. The optimum humidity of the attractive substrate for ovipositing is obtained by the mixing equal parts of the dry substrate and water (1:1).

**Abstract:**

The industrial rearing of *Hermetia illucens* offers sustainable solutions to the acute challenges of modern society associated with the accumulation of increasing amounts of organic waste, the substantial reduction of natural ocean fish stocks, and the imminent food crisis. Detailed knowledge of the reproductive particularities and reproductive behavior of the species is essential for increasing the efficiency of the breeding technology. This study aimed to identify the affinity shown by females regarding the size of the oviposition slots (1, 2, 3, 4 and 5 mm), the vertical distribution of the ovipositing rate, and the influence of the substrate’s moisture on ovipositing behavior (dry matter/water: 1:0.5; 1:1; 1:1.5; 1:2). Over 90% of females oviposited in the 1-, 2- and 3-mm slots, with most of the eggs (58.57%) being oviposited in the 1-mm slots. There was a positive correlation between the size of the oviposition slots and the average weight of the clutches (r = 0.985). The vertical distribution of ovipositing followed a fluctuating trend, with a tendency to oviposit closer to the attractive substrate. The females avoided ovipositing close to substrates with low humidity (1:0.5); indeed, only 6.8% oviposited under these conditions, the differences being significant compared to substrates with higher humidity (*p* < 0.001). The vast majority of females (43.2%) oviposited on substrates with an average humidity of 1:1 (*p* < 0.001). These results provide new insights into the ovipositing behavior of *H. illucens*, which allow for a differentiated harvest of large-sized clutches, providing practical applications with significant impact on the economic efficiency of the species’ industrial growth technology.

## 1. Introduction

The demographic explosion of the last century, associated with an accelerated urbanization, generates an increasing pressure on natural resources and leads to the accumulation of very large amounts of waste, which are increasingly difficult to manage, generating a major impact on the quality of the environment [1,2,3]. These challenges of modern society require finding effective and sustainable solutions to prevent or remedy these shortcomings as much as possible [4]. These aspects substantiated the reorientation of economic policies in all areas towards sustainable circular production systems, associated with a significant reduction of environmental impact, and increasingly involved the scientific community in identifying and capitalizing on opportunities to meet these goals (EU 2018/842) [5].

The growing demand for fishmeal and fish oil in animal husbandry has led to an intensification of oceanic fishing activities, putting undue pressure on the natural resources of ocean fish, significantly reducing natural stocks [6,7,8], which in turn has led to the need to identify alternative sustainable resources. The importance of such alternative resources is increasingly obvious as it is estimated that by 2030 only 10% of fish production will be available for animal husbandry and aquaculture, as 90% is to be used directly for human consumption [9].

In this context, the species *Hermetia illucens* (L.) (Diptera: Stratiomyidae) stands out through the real benefits it offers by its ability to capitalize on a wide range of organic substrates, including manure, achieving fast and efficient waste recycling [10,11,12,13,14,15,16]. The species offers promising prospects in a new field, namely entomoremediation, being useful even in the recycling of plastics and other difficultly biodegradable materials, a sphere of great interest in waste management [17]. Larval biomass obtained by recycling these substrates can be a valuable source of protein, oil and biologically active compounds that can be used in the feeding of farm animals [18,19,20,21,22,23,24]. Encouraging studies have shown that, in some fodders, it can replace fish or soy protein in proportions varying between 25–100%, depending on the species and age category [18,25], and fish oil up to 100% [26]. Although the bioproductive implications of some biologically active compounds and their impact on the quality of animal products and by-products require further studies [27,28,29,30], all these authors recommend the use of *H. illucens* protein meal and oil in the diet of farmed species [31,32,33,34,35,36]. Some qualitative shortcomings of *H. illucens* protein meal and oil can be overcome by manipulating the structure of the larval growth substrate, which can significantly change the nutrient composition of the larvae [37,38,39,40]. The species also offers promising opportunities regarding the use of *H. illucens* oil in the cosmetics industry [41,42], or as a potential source of fuel, biodiesel, respectively [43,44,45,46]. Moreover, organic substrates subjected to larval biocomposting processes can be successfully used as organic fertilizers in horticulture and agriculture [11,47,48]. More detailed studies, however, are needed to assess the microbiological load of processed substrates and to assess the risk of disease transmission to crop plants [49].

There is relatively little data on the most suitable conditions for the maintenance of *H. illucens* breeders, and on specific aspects related to the growth and development of the species, knowledge that is essential for the development of efficient technologies for the valorization of the species [50]. The justified interest for the industrial growth of *H. illucens* [51,52] also implies mastering the fine details related to the species’ reproductive behavior and of the factors with significant impact on this process [53,54].

Studies have shown that the reproductive performances of *H. illucens* are influenced by many factors in the breeding environment, such as temperature [55,56], light [57,58,59], humidity [60], attractive substrate for stimulating ovipositing [50,61,62], ovipositing conditions [63,64], etc. The economic efficiency of the rearing and reproduction of this species is therefore greatly influenced by the most detailed knowledge of the particularities of the reproductive process. Thus, the temperature of the larval growth substrate has been shown to significantly influence the size and lifespan of reproductive adults [65,66]. The size of the females has direct implications on the size and quality of the egg clutches [67]. Clutches with a significantly higher number of eggs were obtained by supplementing the drinking water with a protein source in breeders [68]. Doubling of the lifespan and, implicitly, of the reproductive chances in the reproductive females was obtained by adding sugar in the drinking water [69]. Natural light and its intensity strongly influence the reproductive behavior and ovipositing of *H. illucens*, with a low number of matings and a high incidence of sterile eggs being reported in breeding carried out exclusively under artificial light conditions [70,71]. Moreover, a tendency for females to oviposit primarily in the sun-exposed areas of the ovipositing devices was reported [62]. Volatile compounds from the attractive substrate also play an important role in stimulating ovipositing and in the choice of females for ovipositing sites [72]. These show a differentiated attractiveness towards the nature of the ovipositing devices [73]; indeed, it was found that the number of oviposits on wooden boards and corrugated cardboard is significantly higher than on glass and plastic ovipositing devices [64]. Wooden boards and corrugated cardboard, as ovipositing substrate, are relatively common in the breeding process of *H. illucens* [74,75], but wooden devices appear to be more suitable because they allow individualization and full harvesting of the clutches and do not absorb humidity like the cardboard devices [76].

There is relatively little data on the size of the oviposition slots and even fewer relevant data on the affinity of females for the size of these slots for ovipositing. In the case of both wooden and corrugated cardboard ovipositing devices, the reported size of the laying slots is generally between 3–5 mm [62,63]. If, in the case of corrugated cardboard devices, the size of these slots is fixed, while in the case of wooden plate devices, the oviposition slots are adjustable, obtained by using spacer elements from different materials, metal washers are recommended [76]. Klüber et al. [77] used metal washers to ensure homogeneous 2-mm slots for the ovipositing devices. To our knowledge, no study addresses issues related to the influence of the size of the oviposition slots on the ovipositing rate, namely the specific affinity of females for this parameter. There is also no clear consensus regarding the optimal size of the oviposition slots.

Regarding the distance of ovipositing from the attractive substrate, the data are few and tangential, and there are no studies dedicated specifically to this aspect. Holmes et al. [61] placed corrugated cardboard ovipositing devices at a distance of 5 cm from the substrate, while Park et al. [62] placed the ovipositing devices approximately 3 cm above the substrate. Booth and Sheppard [78] showed that *H. illucens* females tend to oviposit in higher sites when the substrate is excessively wet, an observation that is an interesting premise for the experimental evaluation of the impact of attractive substrate moisture on the ovipositing behavior. This aspect was also suggested by Holmes et al. [74], who showed that the shape, stratification, and weight of the clutches are influenced by the nature and conformation of the ovipositing substrate, mentioning the need for more conclusive data on the attractiveness of females towards ovipositing sites. The usefulness of experiments to assess the ovipositing behavior in correlation with the humidity conditions of ovipositing sites was also emphasized in the conclusions of the report by Tomberlin and Sheppard [70].

In our experiments, we aimed to evaluate the particularities of the ovipositing behavior of *H. illucens* females, from the perspective of their affinity towards the size of the ovipositing slots. We aimed also to identify possible correlations between the quality of the clutches and the size of the ovipositing slots. Other aspects we assessed were the effect of the humidity of the attractive substrate on the ovipositing rate, the characteristics of the clutches and the ovipositing distance from the substrate.

## 2. Materials and Methods

The experiments were carried out at USAMV Cluj-Napoca, Romania, between 12 November 2020 and 22 July 2021.

The colony of *H. illucens* was purchased in 2016 from Greece. It was reproduced in the Ecology and Zoology Laboratory, within USAMV, obtaining 5–6 generations/year. Larval growth was obtained on a standard Gainesville diet [79]. The breeding parameters were maintained within the optimal limits for the species [69]: 27 ± 1 °C; relative humidity of 60 ± 5%; 14 h photoperiod under LED light-6000 lux. The following aspects were observed: (1) the behavioral particularities of the ovipositing process to determine the affinity of females towards various ovipositing slot size; (2) the preferred ovipositing distance from the attractive substrate; (3) the influence of substrate moisture on the ovipositing distance from the substrate and the factors that can be correlated with ovipositing.

**Figure 1:**
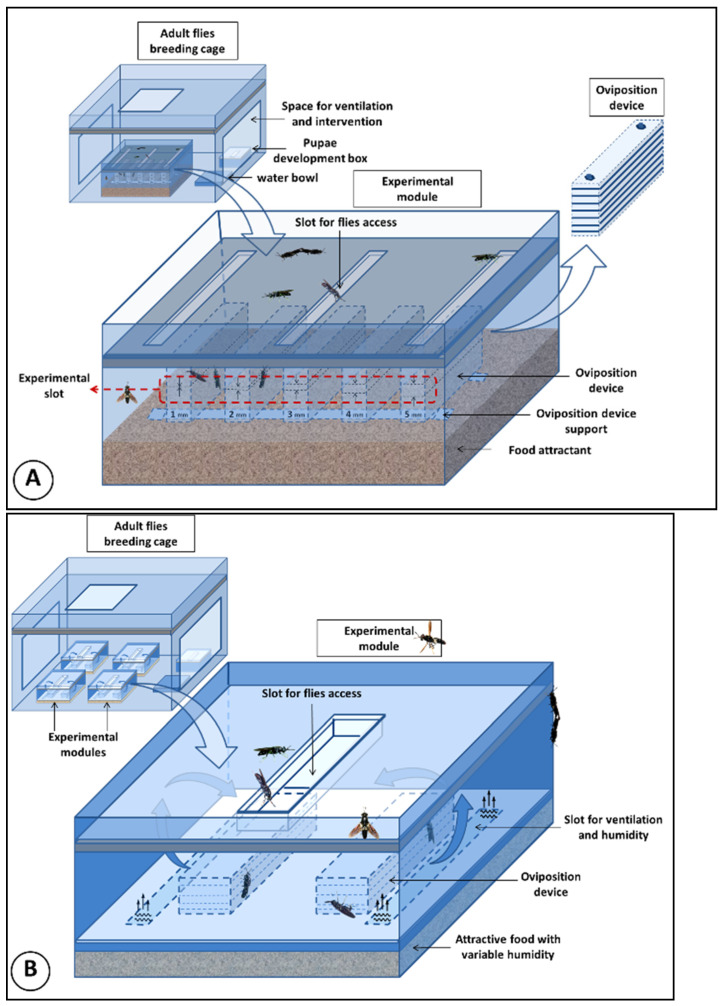
(**A**) Experimental device for evaluating the behavioral peculiarities of laying eggs according to the size of the oviposition slots. (**B**) Experimental device to evaluate the influence of substrate moisture on ovipositing behavior in *Hermetia illucens*.

The experimental breeding enclosure for the breeders consisted of a box with transparent walls 700 mm long × 550 mm wide × 800 mm high, where the reproduction of *H. illucens* and ovipositing took place. On two of the side walls and on the lid, the device was fitted with access windows 400 mm long and 300 mm wide, to which mosquito net sleeves (50 cm long) were tightly attached. These access windows also ensured the ventilation of the experimental enclosure (Figure 1).

In the first experimental stage, in an opaque box 250 mm long × 180 mm wide × 120 mm high, 500 pupae were introduced to complete their development and metamorphosis process. This box was introduced in the experimental enclosure. To determine the preferred size of the ovipositing slots, five ovipositing devices with progressive slot sizes (from 1 to 5 mm) were built. These were made of softwood, in the form of boards 160 mm long × 30 mm wide × 4 mm high. Nine overlapping boards were fixed with transverse screws at both ends. The size of the slot was established using 1 mm thick metal washers [76]. The 5 ovipositing devices were placed in an opaque box 450 mm long × 300 mm wide × 250 mm high. Standard Gainesville-type substrate [79] was beforehand introduced in the ovipositing box as a food attractant. The substrate was periodically wetted to maintain a 60–70% humidity. A support was placed over the substrate on which the ovipositing devices were placed. The box featured an opaque cover with fly access slots. The experimental ovipositing module was in turn introduced in the experimental maintenance enclosure of the breeders (Figure 1). Several studies have mentioned using mosquito nets placed between the attractive substrate and the ovipositing devices, suggesting the tendency of females to oviposit directly on the substrate [59,80]. In our research, to evaluate the predisposition of ovipositing according to the distance from the attractive substrate, we placed the 5 ovipositing devices on two transverse bars buried in the substrate, so that the bottom of the devices was in superficial contact with it (Figure 1).

In the experimental enclosures, the optimal microclimate conditions for this species were ensured: a temperature of 27 ± 1 °C, a humidity of 60 ± 5%, and a lighting regimen of 14 h of light and 10 h of darkness [81]. During the day, natural light was provided and compensated with artificial light during periods of lack of natural light. The artificial light source was white LED light with the 350 to 600 nm wavelength spectrum, the illuminance intensity 6000 lux, brand name: Philips. The ovipositing devices were checked daily, and the clutches were counted, measured, harvested and weighed individually.

In the second experimental stage, which aimed to determine the influence of the humidity of the attractive food substrate on the ovipositing distance, 1000 pupae were placed in an opaque box with the same dimensions as those described above, to complete their development and metamorphosis process. Four experimental modules were introduced in the experimental enclosure described in the first stage. These consisted of opaque boxes 240 mm long × 160 mm wide × 120 mm high (Figure 1).

Inside each box, a plexiglass plate was placed with two ventilation slots covered with a mosquito net. The role of this plate was to prevent the direct contact of the *H. illucens* females with the attractive substrate, but they ensured the dissipation of the substrate moisture inside the experimental module. The ovipositing devices were placed on top of this plate. The box was closed with a lid that had a central slit with the edges turned inwards, through where the females had free access to the ovipositing enclosure (Figure 1). In the four experimental modules, 100 g of Gainesville food attractant [79] were introduced. In group 1, the substrate was moistened with 50 mL of water; in the next three groups, the amount of water increased progressively by 50 mL of water, ensuring a substrate dry matter/water ratio of 1:0.5 in group 1; 1:1 in group 2; 1:1.5 in group 3, and 1:2 in group 3. Two ovipositing devices were introduced in each of the four experimental modules, one of which had 1-mm slots and the other had 2-mm slots. These were checked at 24-h intervals and the number of clutches, the distance of ovipositing from the substrate (measured with an electronic caliper, model Stainless Precise 7215), and the weight of each clutch (by weighing with the analytical balance Explorer-Pro, model EP114C, accuracy 0.01 mg) were recorded. The same microclimate conditions were ensured in the maintenance enclosure as in the previous stage.

### Statistics

The statistical interpretation and graphical representation of the results were performed using Microsoft Excel 2013. Testing the statistical significance of the differences between the number of clutches according to the applied treatments was performed using the nonparametric Mann–Whitney test. The Pearson correlation was used to find the correlations. The differences between the average weights of the clutches, depending on the treatments applied, were tested using one-way ANOVA, followed by the Tukey HSD test. The significance threshold used for all analyses was 0.05.

## 3. Results

Results from the first experimental stage show that the size of the oviposition slots influenced (*p* < 0.001) the number of clutches oviposited and their weight. A total of 140 clutches were counted. Of these, 82 clutches were oviposited in the 1-mm ovipositing device, representing 58.57% of total ovipositing. This was the only slot size with statistically significant differences compared to the others (*p* < 0.05) (Table 1). Although it presented the highest number of clutches, the deposited clutches had the lowest average weight (17.47 ± 0.55 mg, *p* < 0.001) compared to the rest of the ovipositing devices. A number of 23 clutches (16.43%), with an average weight of 21.16 ± 0.83 mg, were deposited in the device with 2-mm slots, and the difference compared to the average weight of the clutches from the 1- and 3-mm slots was significant (*p* < 0.001). The highest average weight of the clutches (26.16 ± 1.04 mg) was recorded in the ovipositing device with 3-mm slots, significantly higher compared to the rest (*p* < 0.001). The ovipositing device with 4-mm slots had the lowest number of clutches, with only three (2.14%), with a mean weight of 23.27 ± 1.13 mg (Table 1).

The clutches were also evaluated from the perspective of the distance from the attractive food substrate. Most of the clutches, 31 (22.14%), were deposited at a distance of 4 mm from the substrate. The number of clutches gradually decreases, with 23 (16.42%) oviposited at 8 mm, and 12 (8.57%) oviposited at a 12 mm distance from the substrate. At 16 mm from the substrate, the number of clutches increases to 22 (15.71%), after which a gradual decrease in the number of clutches is resumed, registering 18 clutches (12.85%) at 20 mm, and only 9 clutches (6, 42%) at 24 mm. A substantial increase in the number of clutches is found at a distance of 28 mm from the substrate, where 25 clutches were recorded, representing 17.85% of the total (Table 1). Significant differences (*p* < 0.05) were found only between the minimum values obtained at a distance of 12 mm (12 clutches), and 24 mm (9 clutches) compared to the number of clutches deposited at all other ovipositing distances (4, 8, 16, 20 and 28 mm, respectively) (Table 1).

The average weight of the clutches varies widely depending on the ovipositing distance from the food substrate; the lowest value of 16.73 ± 0.99 mg was recorded at a distance of 8 mm (Table 1). Relatively close values were recorded in the cases of clutches oviposited at a distance of 4 mm (18.74 ± 0.88 mg), 20 mm (17.83 ± 1.22 mg), and 28 mm (19.78 ± 0.97 mg), although there were no statistical differences (*p* > 0.05). The highest value of the average clutch weight, 25.73 ± 2.03 mg, was recorded at a distance of 12 mm from the substrate, and close average values were seen at distances of 16 mm (24.18 ± 1.26 mg) and 24 mm (22.83 ± 1.55 mg); however, the differences between the three averages were not significant (*p* > 0.05). However, for these three distances (12, 16 and 24 mm), the average weight of the clutches showed significant differences (*p* < 0.001) compared to the other four distances (4, 8, 20 and 28 mm) (Table 1).

In the second experimental stage, it was found that the humidity of the attractive substrate also influences the ovipositing rate. The lowest number of clutches (31) was obtained in group 1 (1:0.5), the differences compared to the number of clutches in all other groups being significant (*p* < 0.05) (Table 2). By contrast, in group 2 (1:1), there were 183 clutches, the highest value recorded in this experiment, the differences from the rest of the groups being significant (*p* < 0.05). Groups 3 (1:1.5) and 4 (1:2) oviposited a close number of clutches, 107 and 103, respectively, with significant differences being recorded only compared to groups 1 and 2 (Table 2).

The use of the two different ovipositing devices, with 1- and 2-mm slots, in the four groups with different humidity levels reconfirmed the results obtained in the first experiment. Thus, in the device with 1-mm slots, 251 clutches were deposited, and in the one with 2-mm slots, 173 clutches were deposited; the difference between the total number of clutches deposited in the two devices was significant (*p* < 0.05) (Table 2).

From the perspective of the ovipositing distance from the attractive substrate, evolutions similar to those recorded in the first experiment were seen. The highest number of clutches was recorded in the immediate proximity of the substrate, namely in the first slot, at a distance of 4 mm, where a total of 129 clutches were counted, regardless of the humidity level of the substrate. It is noteworthy that in both the 1-mm and the 2-mm slot devices, most of clutches were deposited at this distance, with 75 and 54, respectively. The number of clutches gradually decreased to a distance of 16 mm, where only 26 clutches were counted, after which the number of clutches increases to 65 at a distance of 24 mm from the substrate. After this second peak, the number of clutches decreased to 43 at a distance of 28 mm (Table 2).

Significant differences (*p* < 0.05) were recorded between the maximum number of clutches (129 at 4 mm) and all other distances (12, 16, 20 and 28 mm), except for the number of clutches oviposited at a distance of 8 and 24 mm from the substrate. In the device with 1-mm slots, the lowest number of clutches (two) was deposited at a distance of 16 mm from the substrate. In the device with 2-mm slots, the lowest number of clutches (one) was deposited at a distance of 12 mm. Differences from all other numbers of clutches were statistically significant (*p* < 0.05) (Table 2).

The average weight of the clutches deposited in the four experimental groups showed a remarkable homogeneity. Extreme values were identified in group 4 (25.47 ± 0.65 mg) and group 2 (25.92 ± 0.66 mg). There were no significant differences between the average weights of the clutches from the four experimental groups (*p* > 0.05) (Table 3).

The evolution of the average clutch weight depending on the distance of the ovipositing from the attractive substrate varies within relatively wide limits (Table 3). The lowest average clutch weight of 23.64 mg was recorded at a distance of 12 mm, and the highest average clutch weight was 30.41 mg, at a distance of 28 mm. Statistically significant differences were recorded between the average clutch weights at all distances assessed versus the maximum value recorded at a distance of 28 mm (*p* < 0.001). At a distance of 16 mm, the average clutch weight was 27.33 mg, with statistically significant differences compared to all other average clutch weights (*p* < 0.001), except for the weight recorded at a distance of 8 mm (25.25 mg) from which it did not show a statistically significant difference (*p* > 0.05). The highest average clutch weights were registered at a distance of 28 mm regardless of the group (1:0.5, 1:1, 1:1.5, 1:2). The lowest value (29.12 mg) was identified in group 3 (1:1.5) and the highest (32.48 mg) in group 4 (1:2). There were no statistically significant differences between the average weights of clutches recorded at a distance of 28 mm (*p* > 0.05) (Table 3).

The size of the ovipositing slots obviously influences clutch characteristics. In addition to the significantly lower number of clutches deposited in devices with 2-mm slots, the average weight of these clutches was consistently higher in all groups, compared to devices with 1-mm slots. In the latter devices, the lowest average clutch weight (21.06 mg) was recorded in group 1 (1:0.5), and the highest (24.72 mg) in group 2 (1:1) (Table 3). In ovipositing devices with 2-mm slots, the lowest average weight of the clutches (27.40 mg) was recorded in group 3 (1:1.5) and the highest (30.79 mg) in group 1 (1:0.5). The average weight of all the clutches deposited in the devices with slots of 1 mm was 23.06 mg, and of those deposited in devices with slots of 2 mm was 28.63 mg, the difference between the two mean values being statistically significant (*p* < 0.001) (Table 3).

## 4. Discussion

**Figure 2:**
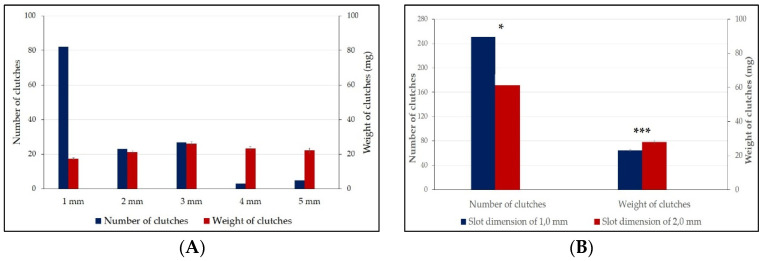
The number of clutches and their average weight depending on the size of the ovipositing slots, in the two experimental stages. (**A**) Experimental stage I; (**B**) experimental stage II. * = *p* < 0.05; *** = *p* < 0.001. Error bars depict standard errors of means.

The nature of the ovipositing device and the size of the slots are important in the manifestation of the *H. illucens* ovipositing behavior. Usually, the ovipositing devices used in various experiments consisted of overlapping layers of corrugated cardboard that showed a relatively wide diversity in terms of the size of the ovipositing slots: 2–3 mm [70], 3–4 mm [74], 2–5 mm [56] and 5 mm [62]. Hoc et al. [76] showed that these devices have a number of disadvantages that can significantly influence the accurate assessment of the real clutch dimensions. The cardboard tends to retain moisture by changing its weight and shape, being able to facilitate multiple ovipositions (several females ovipositing clutches in the same hole) and there are difficulties in harvesting and objective assessment of the weight and number of eggs in the clutch. Situations are also common in which most of the clutches are deposited outside the harvesting devices [59]. These shortcomings are avoided by using removable devices made of wooden boards with adjustable slots [76]. Our experiments confirm the effectiveness of the use in scientific experiments of ovipositing devices made of wooden boards with adjustable slots. Thus, from the very beginning, it should be pointed out that the vast majority of clutches (92%) were deposited in ovipositing devices with slot dimensions of 1, 2 and 3 mm, with slots of similar sizes being used successfully in other experiments [70,77,80]. The free choice of females for ovipositing in the five different ovipositing devices revealed that they showed an obvious preference for the device with 1-mm slots, where most of clutches were deposited (58.57%, *p* < 0.001). However, it is important to point out that these clutches had the lowest average weight, of only 17.47 mg, which gives indications on the small size of the females that show a predisposition for ovipositing in 1-mm slots. This hypothesis is supported by the evolution of ovipositing in the next two devices, where the number of clutches decreases significantly, but their average weight increased (*p* < 0.001) to 21.16 mg in group 2, and to 26.16 mg in group 3. The number of the ovipositions that took place in these last two devices (with 2- and 3-mm slots) accounted for 36.36% of the total. In devices with 4- and 5-mm slots, the number of clutches decreases substantially, to 2.14% and 5.57%, respectively, without affecting the clutches’ weight (*p* > 0.05) (Figure 2).

These results are confirmed by the data obtained in the second experimental stage, which aimed to establish the influence of the humidity of the attractive substrate on the ovipositing rate and the ovipositing distance from it (Figure 2). The impact generated by the humidity of the attractive substrate on the ovipositing behavior was also evaluated from the perspective of the preferred size of the ovipositing slots. As it was established in the previous stage that over 90% of the clutches were deposited in devices with 1–3 mm ovipositing slots, in this experimental stage, only 1- and 2-mm slot devices were used. Evaluation of the number of clutches in the two devices (1 and 2 mm), located in each of the four experimental modules, reconfirmed the affinity of 59.2% of females for the 1-mm slot ovipositing device; the difference compared to the number of clutches in the device with 2-mm slots was statistically significant (*p* < 0.05). This value is very close to that obtained in the first experimental stage, where the number of clutches in the device with 1-mm slots was 58.6%, given that in the second experimental stage, triple the number of clutches were evaluated, with 424 clutches in total (Figure 2). In a previous study, we showed that approximately 65% of the female population included individuals with low and medium weights, and 35% of the female population was represented by plus-variants, weighing more than the average population; these aspects proved to be positively correlated with the weight of the clutches [67].

The ovipositing rate in the 1-mm slot device was 58.64% in the first experimental stage and 59.2% in the second stage, which is close to the percentage of small- and medium-sized females, being 65% of the general population. We believe that small- and medium-sized females, which are the most numerous, find optimal ovipositing conditions in devices with 1-mm slots, and large females show affinity for slots larger than 2 and 3 mm, which correspond optimally to the size of the ovipositor and to the clutch size.

Moreover, in the second experimental stage, the significantly higher average weight of the deposited clutches in the ovipositing device with 2-mm slots (27.87 mg) was reconfirmed, compared to the average weight of the clutches deposited in the device with 1-mm slots, at 23.35 mg (*p* < 0.001). It is noteworthy that this significant difference in the average weight of the clutches from the two ovipositing devices (1 and 2 mm) is confirmed in each of the four experimental modules, regardless of the humidity of the attractive substrate (Figure 2).

The values of the clutch average weights recorded in our experiment are close to those obtained in other experiments that evaluated and recorded this reproductive parameter of the species; for example, 10.57–31.71 mg [70], and 16.6–22, 5 mg [76]. However, the overall average weight of the clutches in our experiment (22.05 mg) was lower than that reported by Booth and Sheppard [78], at 29.1 mg, although similar substrates were used. Conversely, it was much higher than that obtained by Heussler et al. [59], with 1.9–2.3 mg, or by Jucker et al. [82], with 7.81–10.27 mg, probably due to the type of larvae rearing substrate used. The gradual increase in the average weight of the clutches, correlated with the increase in the size of the ovipositing slots, is associated in our opinion with the size of the females. The populations of *H. illucens* show a wide heterogeneity in terms of weight and size of individuals of both sexes [67,81]. It has also been shown that there is a direct correlation between the size of the females and the size of the brood, in the sense that large females have higher energy resources, as reflected in the clutches of heavier weights, and implicitly with a larger number of eggs [67,81].

The evolution of the number of clutches in relation to the distance from the attractive substrate confirms the observations of other authors who recorded the appetite of some females to oviposit directly on the substrate or in its immediate vicinity [59,80]. In the first slot of the ovipositing device, located at 4 mm from the substrate, most clutches were recorded (22.1%). However, the ovipositing rate in the vertical distribution of the ovipositing device seems to follow a fluctuating pattern, on a general downward trend, which suggests that the ovipositing distance from the substrate is not significantly influenced by the actual nature of the Gainesville substrate, but rather the substrate moisture [70]. This aspect is also suggested by Booth and Sheppard [78], who point out that *H. illucens* females tend to oviposit at greater distances from the fresh substrate with the higher humidity.

**Figure 3:**
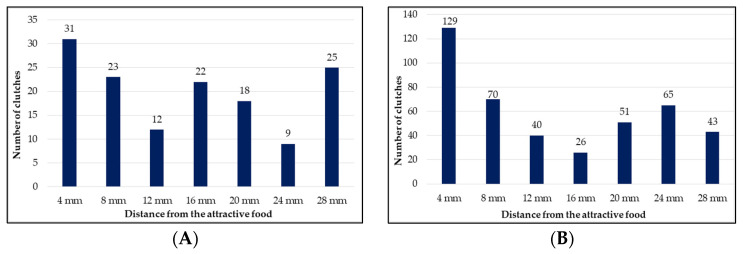
Distribution of the numbers of clutches according to the distance from the substrate in the two experimental stages. (**A**) Stage I, 140 oviposition (N = 500 flies); (**B**) Stage II, 424 oviposition (N = 1000 flies).

The second experimental step aimed to verify these hypotheses regarding the influence of the humidity of the attractive substrate on the ovipositing distance from it. Our results show that most clutches, irrespective of study group, were deposited in the immediate vicinity of the substrate. Indeed, 30.42% of all clutches were deposited in the first slot of the ovipositing device, at a distance of 4 mm from the substrate, and the numerical difference was statistically significant compared to most of the other values recorded at greater distances from the substrate (*p* < 0.05) (Table 2). These data are consistent with the results obtained in the first stage and with the observations of other authors on the predisposition for ovipositing in the vicinity of the substrate [59,80]. The number of clutches gradually decreased to the slot located at a distance of 16 mm, after which it increased again to the slot at 24 mm, the level from which it resumed the downward course following the fluctuating pattern that was described in the previous stage. The ovipositing rate in vertical dynamics follows the downward trend of the number of ovipositions as the distance from the attractive substrate increases (Figure 3).

It should be noted that the evolution of the ovipositing rate depending on the distance from the substrate follows a course similar to that found in the previous experimental stage, where a three-times higher number of clutches were evaluated (424 clutches in the second phase compared with 140 clutches in the first stage) (Figure 3). The similar evolution of the vertical distribution of the number of clutch deposits, irrespective of the humidity of the attractive substrate, does not seem to confirm Booth and Sheppard’s [78] assumption that females of *H. illucens* tend to oviposit at a greater distance from the attractive substrate when it has a higher humidity.

**Figure 4:**
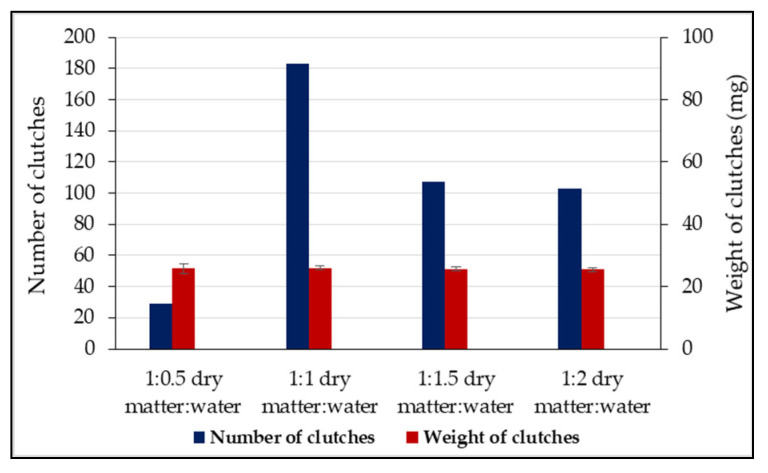
Distribution of the number of clutches (424 egg clutches) and their average weight clutches depending on the humidity of the substrate (Stage II, N = 1000 flies). Error bars depict standard errors of means.

Substrate moisture can substantially influence the rate of *H. illucens* oviposition, so the attractive substrate with low humidity in group 1 (1:0.5) showed the lowest number of clutches, with only 6.8% of females ovipositing in this experimental module; the differences compared to all the other groups were statistically significant (*p* < 0.001). We propose that females avoid substrates with low humidity because it can negatively influence the viability of the eggs and the capacity for embryonic development. According to data reported by Holmes et al. [74], at low atmospheric humidity (25%), dehydration of the eggs increases embryo mortality and causes a slower development of larvae after hatching, effects that can be associated with low humidity of the growing substrate (Figure 4). The general evolution of the number of clutches deposited reveals the significant option of the females for the substrate with a humidity of 1:1 (group 2), where 43.2% of the females oviposited, with significant differences (*p* < 0.001) compared to group 3 (1:1.5), with 25.23% of clutches, and group 4 (1:2), with 24.29% of clutches (Figure 4). The results are within the limits reported by Tomberlin and Sheppard [70], who showed that over 80% of clutches were deposited at an atmospheric humidity of over 60%, largely confirming the evolution of ovipositions in our experiment at a similar growth substrate humidity.

The results of our experiments provide unique information from the perspective of the affinity shown by females towards the size of ovipositing slots, opening new opportunities in streamlining the selection process of *H. illucens* females, which may be the subject of future research. In the context of the sustainable use of biological resources, our research brings a real improvement to the technology of industrial growth of the species because it provides the premises for easy and fast dissociation of large clutches, proven to contain a significantly higher number of eggs [67,70,78]; this also is important for laboratory research. The data obtained show a real potential for improving the industrial growth technology of the species. In this regard, in their conclusion, Jones and Tomberlin [81] showed that, from the point of view of the capitalization of industrial production of this species, it is important to identify an effective method to obtain large-sized individuals of both sexes, because they have a 50% faster growth rate, and higher densities and feed conversion rates [81]. Obtaining large clutches with as many eggs as possible is all the more important, since Liu et al. [80] showed that under indoor conditions (industrial growth), over 50% of eggs are infertile and therefore do not develop into viable larvae [80]. From this perspective, our results have an important practical impact on the efficiency of larval biomass production by ensuring a sustainable mode of production, as it offers the possibility of easy isolation of clutches with larger numbers of eggs, with a greater potential for growth and development, achieving higher and more efficient larval biomass production. The implications are many because the larval masses with the aforementioned productive performances are able to achieve faster and more efficient bioconversion of a wide variety of organic wastes. At the same time, it provides a higher larval biomass, which potentially can be exploited in different areas at lower prices, thus ensuring the economic sustainability of the industrial growth of this species.

## 5. Conclusions

Over 90% of *H. illucens* females oviposited with predilection for ovipositing devices with slots of 1–3 mm.

The differentiated affinity for oviposition in 1-, 2- and 3-mm slots, depending on the size of the females, is positively correlated with the size of the resulting clutches, and allows a differentiated harvesting of large clutches with larger numbers of eggs.

A significant number of females (22–30%) oviposited in the immediate vicinity of the attractive substrate.

The optimum humidity of the attractive substrate for ovipositing is obtained by the mixing equal parts of the Gainesville diet dry matter and water (1:1). At the same time, substrates with low humidity (1:0.5), which significantly reduce ovipositing rates, should be avoided.

We recommend the use of devices with 2- and 3-mm slots that provide heavier clutch weights and the associated productive performances. This is expected to be more economically efficient for the industrial rearing of BSF.

## Figures and Tables

**Table 1:** Number and average weight of the clutches depending on the slot size and the distance from the attractive substrate.

Specification		Distance from the Substrate							TOTAL	
		4 mm	8 mm	12 mm	16 mm	20 mm	24 mm	28 mm	Total Clutches (N = 140)	Clutch Weight (mg)
Slot dimensions	1 mm	18	23	2	0	17	5	13	82 ^a^	17.47 ± 0.55 ^a^
	2 mm	10	0	0	4	1	0	6	23 ^b^	21.16 ± 0.83 ^b^
	3 mm	0	0	7	17	0	3	0	27 ^b^	26.16 ± 1.04 ^c^
	4 mm	2	0	1	0	0	0	0	3 ^b^	23.27 ± 1.13 ^b^
	5 mm	1	0	2	1	0	1	0	5 ^b^	22.17 ± 1.42 ^b^
Total clutches (N = 140)		31 ^a^	23 ^a^	12 ^b^	22 ^a^	18 ^a^	9 ^b^	25 ^a^	Mann–Whitney	F = 18.03 *p* = 0.001
**Clutch weight (mg)**		**18.74 ±** **0.88 ^a^**	**16.73 ±** **0.99 ^a^**	**25.73 ±** **2.03 ^b^**	**24.18 ±** **1.26 ^b^**	**17.83 ±** **1.22 ^a^**	**22.83 ±** **1.55 ^b^**	**19.78 ±** **0.97 ^a^**	**F = 7.145** ***p* = 0.001**	**one-way ANOVA**

Different letters in the same row or column mean significant differences (*p* < 0.05) for the Mann–Whitney test and the one-way ANOVA test. F = calculated value; *p*-value.

**Table 2:** Ovipositing rate according to substrate moisture, slot size (1 mm and 2 mm) and distance to the substrate.

Specification				Distance from the Substrate							Total	
				4 mm	8 mm	12 mm	16 mm	20 mm	24 mm	28 mm	Ʃ Clutches	Ʃ 1 + 2 mm Mann–Whitney
**Ratio dry matter:water**	**1:0.5**	Slot	1 mm	9	1	1	-	3	3	0	**17**	**31 ^a^**
			2 mm	6	2	0	3	2	0	1	**14**	
	**1:1**	Slot	1 mm	35	30	22	0	12	11	13	**123**	**183 ^b^**
			2 mm	14	7	0	16	0	16	7	**60**	
	**1:1.5**	Slot	1 mm	16	12	9	0	12	15	0	**64**	**107 ^c^**
			2 mm	17	3	1	2	0	7	13	**43**	
	**1:2**	Slot	1 mm	15	3	7	2	7	13	0	**47**	**103 ^c^**
			2 mm	17	12	0	3	15	0	9	**56**	
**Total clutches** **Mann–Whitney**			1 mm	75 ^a^	46 ^ab^	39 ^ab^	2 ^c^	34 ^ab^	42 ^ab^	13 ^b^	**251**	**424**
			2 mm	54 ^a^	24 ^ab^	1 ^c^	24 ^ab^	17 ^ab^	23 ^ab^	30 ^ab^	**173**	
			**Ʃ** **1 + 2 mm**	**129 ^a^**	**70 ^ab^**	**40 ^b^**	**26 ^b^**	**51 ^b^**	**65 ^ab^**	**43 ^b^**	**-**	

Different letters in the same row indicate significant differences (*p* < 0.05) for the Mann–Whitney test.

**Table 3:** Influence of substrate moisture and slot size, correlated with ovipositing distance from the substrate, on the clutch weight.

Specification			Clutch Number	Clutch Weight (mg)	The Average Clutch Weight According to the Distance from the Substrate							One-Way ANOVA F = 0.090 *p*-Value 0.966
					4 mm	8 mm	12 mm	16 mm	20 mm	24 mm	28 mm	
**Substrate (DM)/water**	**1:0.5**	**1 mm**	17	21.06 ± 1.88	22.96	26.47	23.10	28.27	24.65	24.57	30.30	**25.76 ± 1.69**
		**2 mm**	14	30.79 ± 2.22								
	**1:1**	**1 mm**	123	24.72 ± 0.84	25.92	24.95	25.53	30.09	22.17	23.03	29.76	**25.92 ± 0.66**
		**2 mm**	60	28.05 ± 1.01								
	**1:1.5**	**1 mm**	64	24.36 ± 0.70	23.64	24.33	23.83	26.59	25.10	26.45	29.12	**25.58 ± 0.62**
		**2 mm**	43	27.40 ± 1.0								
	**1:2**	**1 mm**	47	22.08 ± 0.72	25.64	25.25	22.09	24.36	25.33	23.15	32.48	**25.47 ± 0.65**
		**2 mm**	56	28.31 ± 0.87								
**Clutch weight (mg)** **one-way ANOVA:** F = 5.88 *p* = 0.001					**24.54 ± 6.25 ^a^**	**25.25 ± 6.35 ^ab^**	**23.64 ± 6.27 ^a^**	**27.33 ± 7.52 ^b^**	**24.31 ± 6.96 ^a^**	**24.30 ± 6.57 ^a^**	**30.41 ± 8.37 ^c^**	**25.68 mg**

Different letters indicate significant differences (*p* < 0.05) for the Mann–Whitney test and (*p* < 0.001) for the one-way ANOVA test.

## Data Availability

The data supporting the reported results are in the possession of the authors.
